# Electric field modification of magnetism in Au/La_2/3_Ba_1/3_MnO_3_/Pt device

**DOI:** 10.1038/srep12766

**Published:** 2015-08-04

**Authors:** Y. Q. Xiong, W. P. Zhou, Q. Li, Q. Q. Cao, T. Tang, D. H. Wang, Y. W. Du

**Affiliations:** 1 Jiangsu Key Laboratory for Nano Technology and National Laboratory of Solid State Microstructures, Nanjing University, Nanjing 210093, People’s Republic of China; 2 Collaborative Innovation Center of Advanced Microstructures, Nanjing University, Nanjing 210093, People’s Republic of China

## Abstract

The La_2/3_Ba_1/3_MnO_3_ film is deposited in a CMOS-compatible Pt/Ti/SiO_2_/Si substrate with the oxygen pressure of 10 Pa for investigating magnetoelectric effect. Bipolar resistive switching effect with excellent endurance and retention is observed in this Au/La_2/3_Ba_1/3_MnO_3_/Pt device. Through this effect, a significant nonvolatile change of magnetization is obtained in this device as well. The change of magnetization can be understood by the break and repair of the -Mn^3+^-O^2−^-Mn^4+^- chains induced by the electric field through the oxygen vacancies migration. The resistance and magnetization of the Au/La_2/3_Ba_1/3_MnO_3_/Pt device can be simultaneously manipulated by the electric field, which makes it to be a promising candidate for the multifunctional memory devices.

Manipulation of magnetic properties in nanomaterials with an external electric field is extremely attractive for the promising applications in the next generation information storage, spintronics and low power-consuming magnetoelectric device^1^,^2^,^3^,^4^. Up to now, electric field control of magnetism has been reported in a large number of materials, such as single-phase multiferroic materials^5^,^6^, ferromagnetic/ferroelectric heterostructures^7^,^8^,^9^,^10^,^11^,^12^, and diluted magnetic semiconductors^13^,^14^,^15^,^16^,^17^. However, there are still some problems that remain to be resolved. For example, for the single-phase multiferroic and diluted magnetic semiconductors, most works to date have been carried out only at low temperatures^18^,^19^, which is still some way from the practical applications. In the case of ferromagnetic/ferroelectric heterostructures, a strong magnetoelectric coupling can be realized in these composites at room temperature through strain-mediated mechanism^20^,^21^. In order to produce a strong enough stress, a large electric field is required to be applied on the substrate^22^,^23^, which would be an obstacle for the application. On the other hand, this type of magnetoelectric heterostructures are composed of some special substrates, such as BaTiO_3_, Pb(Mg_1/3_Nb_2/3_)O_3_-PbTiO_3_, or SrTiO_3_^20^,^21^,^22^,^23^,^24^,^25^, which is not compatible with the standard complementary metal-oxide semiconductor (CMOS) process. As mentioned above, an important application for magnetoelectric effect is in the field of information storage. However, in most of magnetoelectric system, the magnetization change cannot be maintained with the electric field switching off^20^,^21^,^22^,^23^,^24^,^25^, which cannot meet the requirement of nonvolatility for the memory device. In this context, the electric field control of nonvolatile magnetization in a CMOS-compatible system at room temperature is greatly desirable.

The doped perovskite manganites R_1−x_A_x_MnO_3_ (R = rare-earth metal; A = divalent element) are a strongly correlated electron system with intrinsic interaction among the charge, spin, orbital, and lattice degree of freedom, displaying a rich spectrum of exotic phenomena^26^. The fantastic electronic and magnetic properties make them to be one of the hottest topics in condensed matter physics. Recently, the electric-field-induced nonvolatile resistive switching (RS) effect in the perovskite manganites has attracted considerable attention due to its physical importance and promising applications in the resistance random access memory (ReRAM) device^27^. At present, the underlying physical mechanism of the RS phenomenon in the perovskite manganites, however, is not yet well known. A common perspective physical origin of this effect is ascribed to the migration of oxygen vacancies (or oxygen ions)^28^,^29^,^30^,^31^,^32^. It is known that the variation of concentration of the oxygen vacancies in the perovskite manganites can lead to not only the change of their transport properties, but also the variation of their magnetic states^33^. Therefore, it may provide an opportunity to achieve the electrical control of nonvolatile magnetization through the RS effect in this strongly correlated system.

La_2/3_Ba_1/3_MnO_3_ (LBMO) is a typical perovskite manganite, which shows colossal magnetoresistance and room-temperature ferromagnetism^34^,^35^. It has been reported that the resistance of LBMO can be enhanced by more than 4 orders of magnitude at room temperature while the oxygen content is reduced by ~5%^33^. Meanwhile, the corresponding change of magnetization is also observed due to the variation of oxygen content. Based on this interesting property, we think that LBMO would be the good candidate for investigating the electric field control of magnetization at room temperature through RS effect. In this letter, we deposit the LBMO film on a CMOS-compatible Pt/Ti/SiO_2_/Si substrate and observe the electric field manipulation of nonvolatile change of resistance and magnetization in this system.

## Results

**Figure 1:**
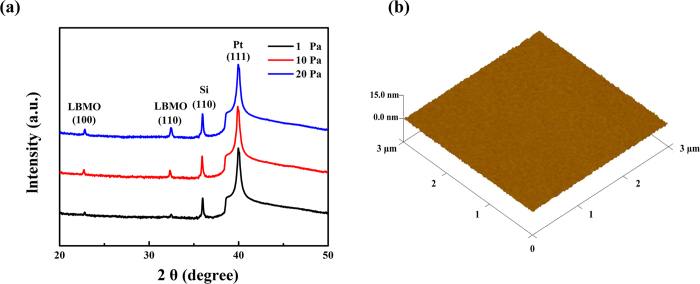
(**a**) X-ray diffraction patterns of the LBMO films deposited on the Pt/Ti/SiO_2_/Si substrates. (**b**) The surface morphology of the LBMO film deposited under 10 Pa oxygen pressure with an area of 3 × 3 μm^2^.

Figure 1(a) shows typical XRD patterns for the LBMO thin films prepared on the (111)-oriented platinized silicon substrates under different oxygen pressures. Two peaks of (100) and (110) are observed, indicating the polycrystalline nature of the films. Figure 1(b) shows the surface morphology of the LBMO film deposited under 10 Pa. The surface roughness of the film is less than 2 nm, which can also be observed in the films deposited under 1 and 20 Pa (not shown).

**Figure 2:**
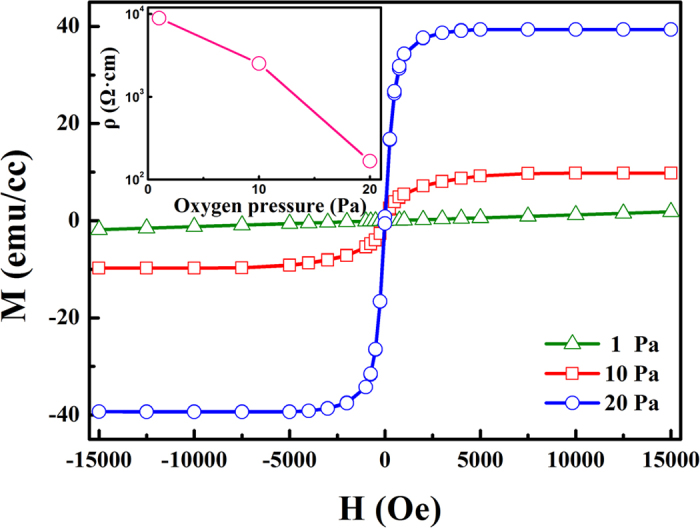
Room-temperature magnetic hysteresis loops of LBMO films deposited at different oxygen pressures. The inset provides the resistivity of LBMO films prepared under different oxygen pressures.

In order to investigate the influence of oxygen content on the magnetic and electronic properties of LBMO films, the magnetization and resistivity of three samples deposited under different oxygen pressure are measured. Figure 2 presents the magnetization versus magnetic field (M-H) curves of these LBMO films at room temperature. During the measurement, the magnetic field with a maximum value of 1.5 T is applied parallel to the surface of the films. Here the diamagnetism signals of the substrates have been deducted. It can be seen that the films deposited under 10 Pa and 20 Pa exhibit typical ferromagnetic behavior with different saturated magnetization (M_s_). For the film grown under 1 Pa, a paramagnetic behavior is observed. The magnetization of LBMO films increases monotonically with increasing oxygen pressure. As shown in the inset of Fig. 2, the resistivity of LBMO films show an opposite tendency with that of magnetization: it decreases as the oxygen pressure increases. The same results have also been reported in the films of La_1-x_Sr_x_MnO_z_^36^, and La_0.67_Ca_0.33_MnO_z_^37^, suggesting that the oxygen content plays a key role in determining the magnetic and electric properties of perovskite manganite films.

**Figure 3:**
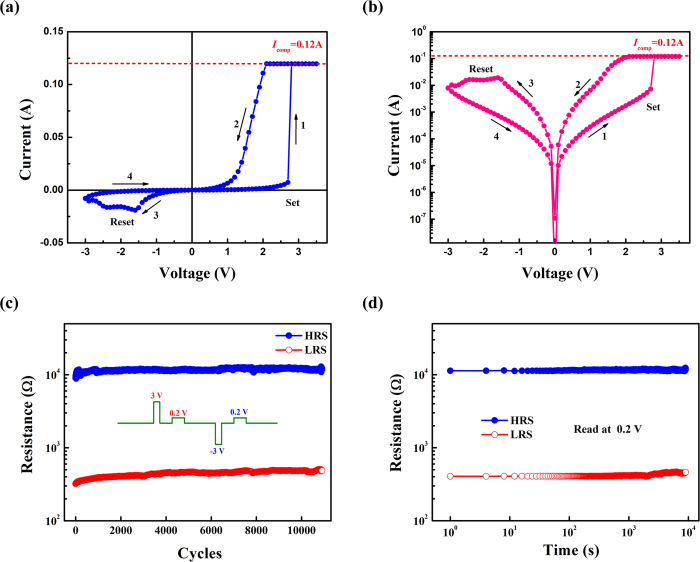
(**a**) Typical bipolar resistive switching characteristic of the Au/LBMO/Pt device. (**b**) I–V curve on a semi-log scale of RS characteristic of the Au/LBMO/Pt device. (**c**) The endurance property of the Au/LBMO/Pt device as a function of switching cycles. The inset of (**c**) illustrates the HRS/LRS switching mode dependence of the evolution of Voltage pulse. The read voltage is 0.2 V (**d**) The retention property of the Au/LBMO/Pt device as a function of time.

In this work, we choose the film deposited under 10 Pa oxygen pressure to investigate the effect of electric field on resistance and magnetism. The reasons are as following: first, the resistivity of the film grown under 20 Pa is too small and the stable RS effect cannot be observed in this sample (not shown); second, for the film deposited under 1 Pa, the stable RS effect can be obtained but the ferromagnetic behavior disappears. Therefore, the film grown under 10 Pa is the very one for our study due to the coexistence of large resistivity and ferromagnetism. Figure 3(a) shows the I–V characteristics of the Au/LBMO/Pt device under a direct voltage sweeping mode, in which the voltage is applied on the top Au electrode while the Pt bottom electrode is grounded. Here the voltage bias is scanned as following sequence: 0 V → +3 V → 0 V → −3 V → 0 V. It should be noted here that a Forming process is not required for the device. With an increase of voltage from 0 V, it can be seen that a set process occurs, which triggers an abrupt change from a high resistance state (HRS) to a low resistance state (LRS). The device can keep at LRS sweeping from 3 V to 0 V. During the set process, a current compliance (*I*_comp_) of 120 mA is adopted to avoid permanent dielectric breakdown of the device. By sweeping applied voltage from 0 V to −3 V, corresponding to a reset process, a converse resistive transition from LRS to HRS occurs. Figure 3(b) presents the hysteretic I–V curve in semi-log scale. The arrows and numbers in the figure indicate the bias voltage sweeping direction and sequence. As illustrated in the I–V curve, the Au/LBMO/Pt device exhibits a typical bipolar RS characteristic, in which the film switches from HRS to LRS in a positive bias region and vice versa in a negative bias region.

Reliability is an important factor for the application of the ReRAM device. Here, both endurance and retention characteristics of the Au/LBMO/Pt device have been investigated. Electric pulses of +3 V and −3 V are alternatively applied on the device, and the resistance of this device was measured at 0.2 V between two neighboring pulses. One cycle of the voltage pulse utilized for the device programming is shown in the inset of Fig. 3(c). The pulse cycle dependence of the resistance for the device is shown in Fig. 3(c), in which two distinct states of high resistance and low resistance can be easily distinguished. The resistance ratio of the device is about 30, which can meet the requirement of the resistance ratio (10 times) for nonvolatile resistive memory application. The two stable resistance states can be maintained up to more than 10^4^ cycles. Figure 3(d) displays the retention characteristic of the Au/LBMO/Pt device, which is obtained at a read voltage of 0.2 V. The resistance values of both HRS and LRS can remain stable up to 10^4^ s without any obvious degradation. All these results reveal that the RS effect in the Au/LBMO/Pt device is repeatable and reliable, which provides a favorable condition for the following magnetoelectric coupling measurements.

**Figure 4:**
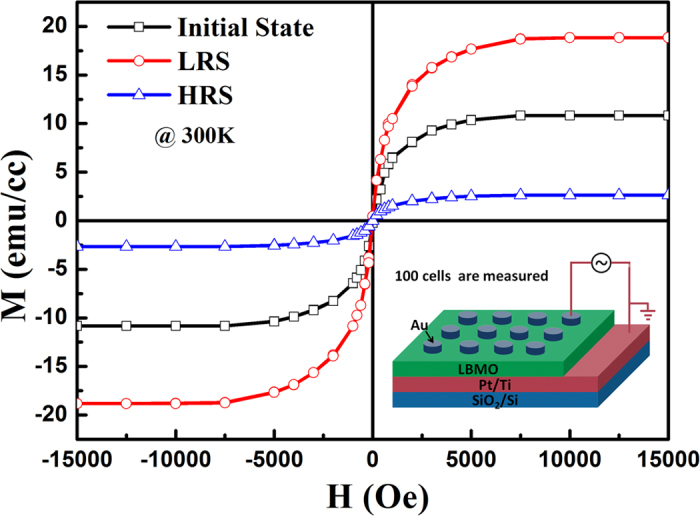
Room-temperature magnetic hysteresis loops of the Au/LBMO/Pt device for the initial state, LRS and HRS. The inset of Fig. 4 is the schematic diagram of Au/LBMO/Pt memory cell.

As mentioned above, LBMO is a strongly correlated electronic system, in which the change of electric property would lead to the variation of magnetism. Therefore, it is necessary to investigate the effect of RS behavior on the magnetic properties of the LBMO film. In our work, only the magnetization of the LBMO film covered by the Au top electrode can be changed with the RS effect. In order to obtain enough clear magnetic signal through RS effect, 100 memory cells with 100 μm-diameter on the 3 mm × 3 mm samples are used for magnetic measurement, which is shown in the inset of Fig. 4. Here we define the resistive state of as-prepared LBMO film as the initial state. Figure 4 shows the magnetic hysteresis loops for the LBMO film in different resistive states at room temperature. Three typical ferromagnetic curves with different M_s_ are observed. In the initial state, M_s_ for the as-prepared film is 10.82 emu/cc. After triggering the memory cells to the LRS state, the value of M_s_ significantly increases to 18.83 emu/cc. When the device is switched back to the HRS state, a reduced value of M_s_ of 2.64 emu/cc is observed. Obviously, the magnetization varies with the different resistive states caused by the electric field, indicating a magnetoelectric coupling effect in the Au/LBMO/Pt device.

**Figure 5:**
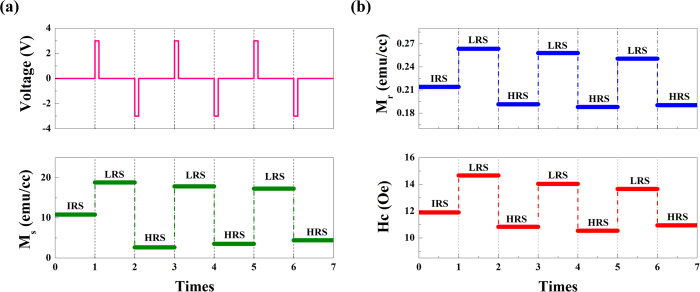
(**a**) The reversible and nonvolatile change of saturation magnetization for the Au/LBMO/Pt device during the reset and set processes. (**b**) Reversible change of the M_r_ and H_c_ accompanying the RS effect.

For the information storage point of view, the reversibility and nonvolatility of memory state is of great importance. To investigate the reversibility of the magnetization in the Au/LBMO/Pt device, we measure the magnetic hysteresis loops in the LRS and HRS for several cycles. The variation of M_s_ for the LBMO film as a function of voltage is shown in the Fig. 5(a). M_s_ of the LBMO film shows almost stable change after several cycles with the voltage triggering back and forth, suggesting that the magnetization of this device can be reversibly and reproducibly controlled by the electric field. Moreover, unlike the effect of electric field control of magnetization in some magnetoelectric composites^20^,^21^,^22^,^23^,^24^,^25^,^38^,^39^,^40^, the magnetization of the memory cells maintains unchanged by switching off the voltage, showing a nonvolatile behavior. When the sample is triggered by a set voltage of 3 V, M_s_ of the film increases to 18.83 emu/cc and keeps constant until a reset voltage of −3 V is triggered again. Furthermore, it is worth noting that the nonvolatile changes of resistance and magnetization can be simultaneously realized in this device by applying electric field, which makes it possible for the integration of stable magnetic and electric signals into one simple device. Reversible change of the residual magnetism (M_r_) and coercive field (H_c_) can be also obversed during the RS effect in the Fig. 5(b), which confirms the RS-induced magnetic modulation in LBMO film.

## Discussion

The mechanism of the RS effect in the perovskite manganites is interesting but still controversial. Several models have been proposed to explain the physical origin of this phenomenon, such as a redox of top electrode^41^, change of schottky-like barrier with electrochemical migration^42^, redox of Metal-O-Metal conducting chain^29^, or Mott metal-insulator transitions^43^. However, increasing evidence shows that the motion of oxygen ions or vacancies in the vicinity of the electrode area plays an important role for the resistance change in perovskite manganites^28^,^29^,^30^,^31^,^32^,^44^,^45^. It has been reported that the RS effect of the perovskite manganite films stems from the break or repair of the -Mn^3+^-O^2−^-Mn^4+^- chains induced by the electric field through the oxygen ions or vacancies migration^29^,^46^,^47^. Here we attempt to use this mechanism to understand the electric field manipulation of magnetism in the Au/LBMO/Pt heterostructure. When a positive voltage is applied on the top electrode, the oxygen ions would migrate toward the vacancies located near the top metal electrode interface and repair the broken chains of -Mn^3+^-O^2−^-Mn^4+^-. On the contrary, a sufficient negative voltage would move oxygen vacancies into the interface region, and piling them up at the metal interface, thereby breaking the -Mn^3+^-O^2−^-Mn^4+^- chains. Therefore, the migration of oxygen ions or vacancies caused by the electric field can result in the break or repair of the -Mn^3+^-O^2−^-Mn^4+^- chains in perovskite manganite film near the top metal electrode interface. As we know, the double exchange interaction between the pairs of Mn^3+^ and Mn^4+^ is responsible for the ferromagnetism of LBMO film^22^. When the -Mn^3+^-O^2−^-Mn^4+^- chains are repaired, it would enhance the exchange interaction, resulting in a high magnetic state. On the contrary, the break of the chains would give rise to a low magnetic state due to the weakening of double exchange interaction. During the set and reset processes, the dynamic current density is about 10^2^ to 10^3^ A/cm^2^, which may play a critical role in oxygen ions or vacancies movement in the interface region of the LBMO film. It has been reported that high current densities can enhanced the diffusion of oxygen in oxides^48^,^49^. Thus in this oxygen diffusion model, the oxygen ion-vacancy concentration can be locally changed at the metal interface by the electric field, which leads to the transformations on the transport and magnetic properties of the LBMO film. Here, it should be mentioned that the complete mechanism of the RS effect might be more complicated due to the strong coupling between electronic, spin, orbital, and elastic degrees of freedom in these complex oxides. More works needs to be done to confirm this oxygen diffusion model overcoming the difficulties of direct measurement of vacancy concentration at the nanometer scale.

In summary, a bipolar resistive switching phenomenon with excellent endurance and retention has been observed in an Au/LBMO/Pt device. Through this effect, the electric field manipulation of nonvolatile magnetization is achieved in this heterostructure, which can be understood by the break or repair of the -Mn^3+^-O^2−^-Mn^4+^- chains induced by the electric field through the oxygen vacancies migration. Moreover, the fabrication of this device is fully compatible with the standard CMOS process. All these good features make the Au/LBMO/Pt heterostructure have a promising future for designing the multifunctional memory devices.

## Methods

LBMO thin films were deposited on the Pt/Ti/SiO2/Si substrates by pulsed laser deposition (PLD) from the stoichiometric target. Deposition was carried out using a 248 nm KrF excimer laser at 3 Hz with a substrate temperature of 650 °C and the oxygen pressure ranged from 1 to 20 pa. The thickness of LBMO films was estimated to be about 350 nm according to the growth rate. Circular Au top electrodes with diameter of 100 μm were deposited using a shadow mask in an ion sputtering instrument. The crystal structure of LBMO film was investigated by X-ray diffraction (XRD) with Cu Kα radiation. The surface morphology of the film was obtained using an atomic force microscope (SPM, Veeco Dimension V). The electric measurement was performed using Keithley 2400 system in an air environment at room temperature. During the measurement, positive bias voltage was applied on the top electrode while the bottom electrode was grounded. The magnetic properties were measured with a superconducting quantum interference devices magnetometer (SQUID, Quantum Design).

## Additional Information

**How to cite this article**: Xiong, Y. Q. *et al*. Electric field modification of magnetism in Au/La_2/3_Ba_1/3_MnO_3_/Pt device. *Sci. Rep.* **5**, 12766; doi: 10.1038/srep12766 (2015).
